# Transcription factor NFAT1 controls allergic contact hypersensitivity through regulation of activation induced cell death program

**DOI:** 10.1038/srep19453

**Published:** 2016-01-18

**Authors:** Ho-Keun Kwon, Gi-Cheon Kim, Ji Sun Hwang, Young Kim, Chang-Suk Chae, Jong Hee Nam, Chang-Duk Jun, Dipayan Rudra, Charles D. Surh, Sin-Hyeog Im

**Affiliations:** 1 School of Life Sciences, Gwangju Institute of Science and Technology (GIST), 123 Cheomdan-gwagiro, Buk-gu, Gwangju, 500-712, Republic of Korea; 2 Academy of Immunology and Microbiology (AIM), Institute for Basic Science (IBS), Pohang, 790-784, Republic of Korea; 3 New Drug Development center, Daegu Gyeongbuk Medical Innovation Foundation, 80, Cheombok-ro, Dong-gu, Daegu, Korea; 4 Chonnam National University Medical School, Gwangju 501-749, Korea; 5 Division of Integrative Biosciences and Biotechnology (IBB), Pohang University of Science and Technology, Pohang, 790-784, Republic of Korea

## Abstract

Allergic contact hypersensitivity (CHS) is an inflammatory skin disease mediated by allergen specific T cells. In this study, we investigated the role of transcription factor NFAT1 in the pathogenesis of contact hypersensitivity. NFAT1 knock out (KO) mice spontaneously developed CHS-like skin inflammation in old age. Healthy young NFAT1 KO mice displayed enhanced susceptibility to hapten-induced CHS. Both CD4^+^ and CD8^+^ T cells from NFAT1 KO mice displayed hyper-activated properties and produced significantly enhanced levels of inflammatory T helper 1(Th1)/Th17 type cytokines. NFAT1 KO T cells were more resistant to activation induced cell death (AICD), and regulatory T cells derived from these mice showed a partial defect in their suppressor activity. NFAT1 KO T cells displayed a reduced expression of apoptosis associated BCL-2/BH3 family members. Ectopic expression of NFAT1 restored the AICD defect in NFAT1 KO T cells and increased AICD in normal T cells. Recipient Rag2^−/−^ mice transferred with NFAT1 KO T cells showed more severe CHS sensitivity due to a defect in activation induced hapten-reactive T cell apoptosis. Collectively, our results suggest the NFAT1 plays a pivotal role as a genetic switch in CD4^+^/CD8^+^ T cell tolerance by regulating AICD process in the T cell mediated skin inflammation.

Allergic contact hypersensitivity (CHS) or allergic contact dermatitis is an inflammatory skin disease mediated by antigen specific T cells. Various allergens including UV, poisons, chemicals and irritants direct the development and progression of CHS^1^,^2^. CHS is considered to be a T helper 1 (Th1)/Th17-associated inflammatory skin disorder^3^,^4^, which can be induced by topical application of a hapten, a small molecule that can elicit an immune response only when attached to a large carrier such as a protein. Upon sensitization by haptens, tissue residual Langerhans cells capture, process and present haptenated antigens (Ags) to T cells to generate hapten specific CD4^+^ and CD8^+^ T cells. Exposure to the same hapten leads to rapid migration of hapten-specific T cells into inflamed tissues to induce severe inflammation by producing large amounts of pro-inflammatory cytokines and cytotoxic effector molecules^4^. Both CD4^+^ and CD8^+^ T cells mediate development and progression of CHS. Hapten-specific CD4^+^ T cells mainly produce inflammatory cytokines (IFNγ and TNFα), which leads to the activation of resident immune cells at the inflamed site^3^,^5^,^6^. Hapten-specific CD8^+^ T cells induce hyper-cytotoxic T lymphocyte (CTL) responses by producing pro-inflammatory (IL17, IFNγ and TNFα) and cytolytic molecules (perforin and granzymes), resulting in massive apoptosis of keratinocytes^3^. Although major pathological significance of cell types and effector molecules are well defined, the roles of transcription factors and their down-stream target genes involved in CHS pathogenesis remain poorly understood^7^.

The Ca^2+^/calcineurin signaling pathway is involved in various biological processes and one of its most well characterized downstream targets, the nuclear factor of activated T cells (NFAT)^8^ is a prominent transcription factor that plays crucial roles in diverse immune functions^9^. NFAT family of transcription factors is composed of five proteins (NFAT1 through NFAT5 (TonEBP)). Among them, NFAT1 to NFAT4 are regulated by Ca^2+^/calcineurin pathway, from which NFAT1 (NFATc2), NFAT2 (NFATc1) and NFAT4 (NFATc3) are mainly expressed in the immune cells^9^. In T lymphocytes, NFAT1 regulates both immunity and tolerance depending on its associated partners^9^. For immunity, NFAT1 plays a key role in mediating T cell activation^10^,^11^, cell cycle^12^,^13^ and differentiation of T helper cells including Th1/Th2^14^, Th17^15^,^16^ and follicular T helper cells^17^. NFAT1 may regulate activation-induced cell death (AICD) program in T cells by up-regulating the expression of Fas ligand (CD95) by directly binding to its promoter region^18^,^19^. Although a potential role of NFAT proteins, in particular NFAT1, in skin inflammation has been suggested^20^,^21^, the role of NFAT1 in contact hypersensitivity skin inflammation and the underlying mechanism of its action remains unclear. However, functional importance of NFAT proteins in regulatory T cells is still not clear. NFAT proteins are required at different stages throughout Treg cell lifespan and have been implicated as a key component of the Treg cell specific transcriptional machinery critical for their optimal function and homeostasis. Binding of Smad3 in cooperation with NFAT2 to a conserved noncoding sequence CNS1 of *Foxp3* drives TGFß mediated extrathymic generation of Treg cells^22^,^23^. Interaction between Foxp3 with NFAT1 has been observed in proteomic analyses of Foxp3-interactome^24^. Structural studies demonstrate the existence of a ternary complex between NFAT, FOXP3 and a DNA element representing *Il2* promoter sequence where NFAT1 associates with a domain swapped dimer of Foxp3^25^. Furthermore, Treg specific deletion of calcineurin B1 (Cnb1) in mice, leading to loss of NFAT activation and nuclear translocation, results in impaired Treg function and severe autoimmunity^26^. More recently NFAT2 and NFAT1 have been demonstrated to bind *Foxp3*’s CNS2 region upon TCR stimulation, thereby promoting interaction between Foxp3 promoter and CNS2, and eventually leading to homeostatic maintenance of Foxp3’s expression and Treg cell identity under inflammatory conditions^27^.

In this study, we have investigated the pathophysiological roles of NFAT1 in T cell-mediated experimental contact hypersensitivity. We found that NFAT1 KO mice spontaneously developed skin inflammation in old age, and healthy young NFAT1 KO mice were more susceptible to hapten induced allergic contact hypersensitivity. Enhanced CHS susceptibility of NFAT1 KO mice was closely related with a functional defect in AICD in both CD4^+^ and CD8^+^ T cells due to down-regulation of apoptosis mediators such as FasL and pro-apoptotic Bcl-2/BH3 family proteins. In addition, NFAT1 deficient CD4^+^ and CD8^+^ T cells produced higher levels of pro-inflammatory and cytolytic molecules, respectively, resulting in exacerbated CHS progression.

## Results

### Spontaneous development of skin inflammation in NFAT1 deficient mice

**Figure 1:**
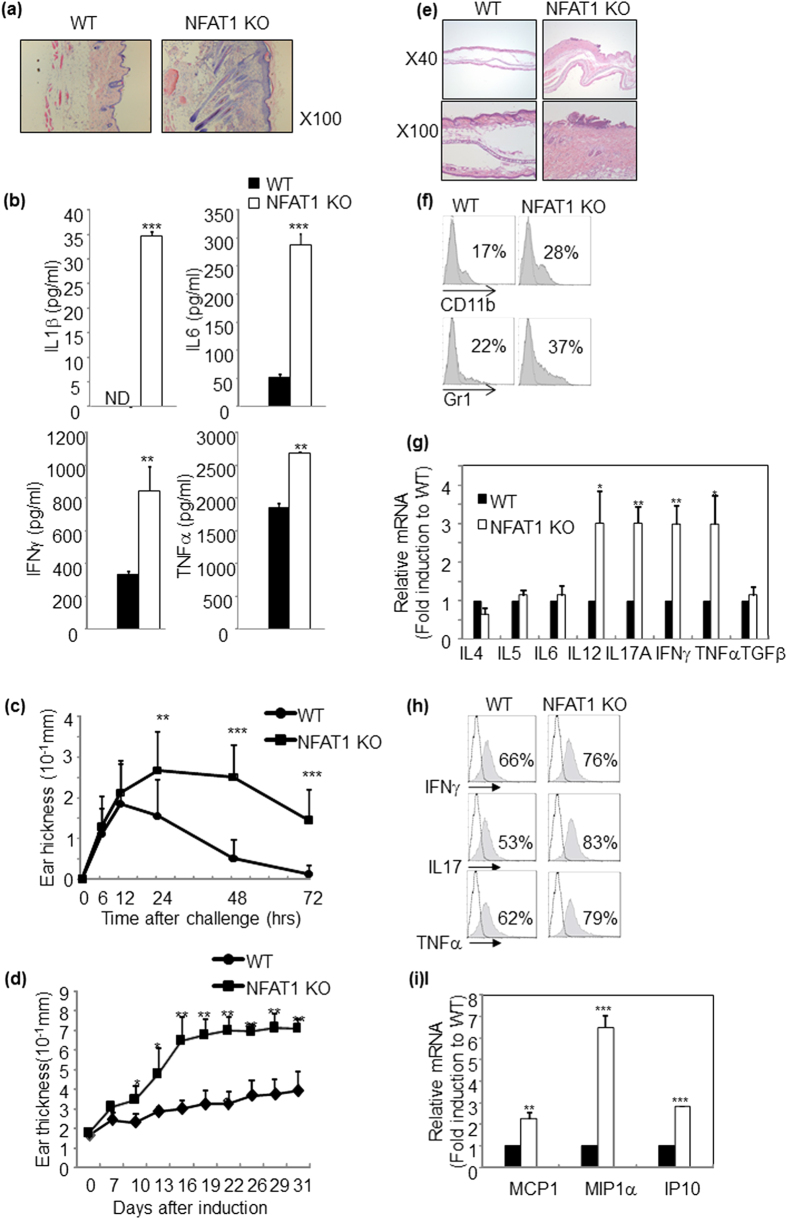
NFAT1 deficiency leads to development of skin inflammation. (**a**) Histological analysis of inflamed lesion from NFAT1 KO (>40 weeks old) and age matched WT mice by H&E staining. (**b**) Pro-inflammatory cytokine levels in serum as determined by ELISA. 10 mice per group; error bars indicate SD. (**c,d**) CHS was induced in young (6 weeks old) mice of indicated genotypes and ear thickness was monitored at different time points after acute phase (**c**) or chronic stage (**d**) of CHS induction. (**e**) Histological analysis was performed upon H&E staining with ear lesions after inducing chronic CHS. (**f**) Analysis of ear infiltrated immune cells by FACS. Dashed histogram is isotype control. The level of cytokines (**g,h**) or chemokines (**i**) expression in ear infiltrated lymphocytes was analyzed by qRT-PCR or flow cytometry n = 5–10 mice per group. Experiments were repeated at least three times. Data are average of independent experiments; error bars indicate SD. *p < 0.05, **p < 0.005 and ***p < 0.001.

Transcription factor NFAT1 plays a key role in development and function of immune system by regulating various lymphokines and anergy-associated genes^28^. However, pathophysiological role of NFAT1 especially in inflammatory skin disorders is still unclear. Interestingly, we found that older (>40 weeks of age) NFAT1 KO mice spontaneously developed mild symptoms of skin inflammation under conventional housing condition (Fig. 1a). These mice displayed edema mainly in their dorsal skin with increased epidermal thickness and infiltration of mononuclear cells, while age-matched wild type (WT) mice did not show any of those symptoms (Fig. 1a). NFAT1 KO mice also showed significantly higher levels of pro-inflammatory cytokines^7^ in their serum, such as IL1β, IL6, TNFα and IFNγ (Fig. 1b). Consistently, compared with WT littermate, CD4^+^ T cells from aged NFAT1 KO mice produced increased level of IFNγ and IL17 but not IL4 (Supplementary Fig. S1). These observations indicated that NFAT1 may regulate development of Th1/Th17 type skin inflammation.

In order to investigate the functional importance of NFAT1 in development of skin inflammation, we measured susceptibility of young NFAT1 KO mice to experimental allergic contact hypersensitivity (CHS). Both acute and chronic models of experimental CHS were induced in healthy WT and NFAT1 KO mice by topical application of a hapten DNCB onto the ears. In the acute model of CHS (aCHS), WT mice completely recovered from an acute inflammation of the ear within 3 d, while NFAT1 KO mice showed a stronger and longer sustained inflammation of the ear that only partially resolved in 3 d (Fig. 1c). The mice were next tested on the chronic model of CHS (cCHS), which is induced by repeatedly painting the ears with DNCB once in 4–5 d intervals. WT mice showed a gradual and marginal increase in ear thickness, while NFAT1 KO mice displayed a significantly faster kinetics of increase in ear swelling that plateaued to a much higher ear thickness and lasted till the end of the experiment (Fig. 1d). NFAT1 KO mice also showed severe signs of inflammation, such as malformation of ear and edema that was confirmed histologically. Hence, compared with WT, NFAT1 KO ears showed significantly increased epidermal- and dermal-thickness accompanied by drastic infiltration of lymphocytes and granulocytes (Fig. 1e). Consistent with histological analysis, significant enrichment of CD11b^+^ macrophages and Gr1^+^ granulocytes was observed in the ears of KO mice compared to WT counterparts (Fig. 1f). These results indicate that NFAT1 may regulate development and progression of T cell-mediated contact hypersensitivity.

### NFAT1 deficiency up-regulates pathogenic effector molecules

**Figure 2:**
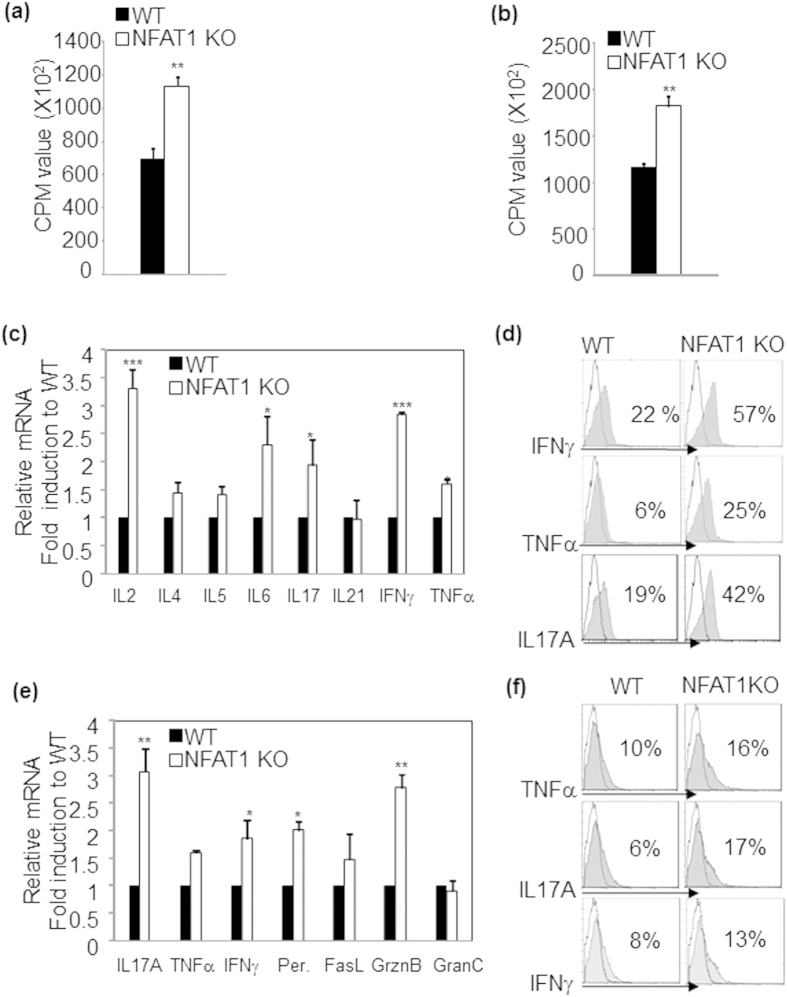
Up-regulation of pathogenic effector molecules in CHS induced NFAT1 KO mice. CD4^+^ or CD8^+^ T cells were obtained from draining lymph nodes of mice with chronic stage of CHS and hapten specific proliferation of CD4^+^ (**a**) and CD8^+^ T cells (**b**) was measured by [H^3^]-thymidine incorporation assay. The levels of cytokine expression in CD4^+^ (**c**) or CD8^+^ T cells (**e**) were analyzed by qRT- PCR. Cytokine expression in WT mice was set at 100%. Flow cytometry analysis was performed to measure cytokine production in CD4^+^ (**d**) or CD8^+^ T cells (**f**). *n* = 5–10 mice per group. Experiments were repeated at least three times. Data are the average of independent experiments; error bars indicate SD. *p < 0.05, **p < 0.005 and ***p < 0.001.

To identify the effector molecules involved in enhanced CHS susceptibility in NFAT1 KO mice, we measured the expression levels of diverse cytokines during the chronic stage of CHS. Total viable cells obtained from the inflamed ear lesion of WT and NFAT1 KO mice were stimulated with PMA and ionomycin, and synthesis of pathogenic pro-inflammatory cytokines^7^ (IL12, IL17, IFNγ and TNFα) and Th2 type cytokines (IL4 and IL5) were measured by RT-PCR or intracellular cytokine staining. NFAT1 KO cells produced significantly higher levels of IL12, IL17, IFNγ and TNFα both at the mRNA (Fig. 1g) and protein (Fig. 1h) than WT cells. No difference was observed in the expression level of Th2 cytokines between the two groups (Fig. 1g). NFAT1 KO cells also produced significantly increased levels of chemoattractants, MCP1, MIP1α and IP10 that mediate the pathogenesis of contact hypersensitivity at the site of inflammation than WT cells (Fig. 1i). We also measured the proliferation and expression levels of major effector molecules of CHS pathogenesis in T lymphocytes. CD4^+^ or CD8^+^ T cells isolated from lymph node of mice at the chronic stage of CHS were stimulated with T cell-depleted splenocytes treated with mitomycin C, and hapten-specific proliferation was measured by [H^3^]-thymidine incorporation assay. Both CD4^+^ (Fig. 2a) and CD8^+^ (Fig. 2b) T cells from NFAT1 KO mice displayed enhanced proliferation compared to those from WT mice. Moreover, compared with WT counterparts, CD4^+^ T cells from NFAT1 KO mice produced significantly increased levels of pro-inflammatory cytokines as analyzed by mRNA (Fig. 2c) and protein (Fig. 2d) levels. Likewise, CD8^+^ T cells from NFAT1 KO mice synthesized significantly higher amounts of IL17A, IFNγ, perforin (Per), and granzymes B (GrznB) (Fig. 2e,f). Collectively, these data indicate that elevated levels of pathogenic effector molecules were produced by T cells in inflamed tissues of NFAT1 KO mice in a fashion that is consistent with exacerbated CHS symptoms observed in these mice.

### NFAT1 deficiency down-regulates the suppressor activity of Foxp3^+^ Treg cells

**Figure 3:**
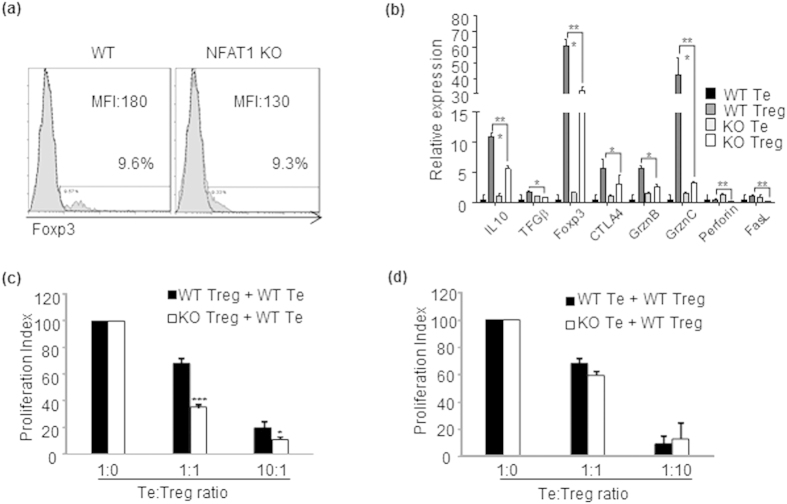
Compromised suppressor activity of NFAT1 deficient Treg cells. (**a**) Foxp3^+^ Treg population was compared by flow cytometry in CD4^+^ T cells isolated from WT and NFAT1 KO mice. (**b**) Expression levels of Treg-associated molecules were measured by qRT-PCR in sorted CD4^+^CD25^−^ (effector; Te) and CD4^+^CD25^+^ (Treg) T cells from WT (WT Te) and NFAT1 KO (KO Te) mice. (**c**) CD4^+^CD25^+^ Treg cells from WT (WT Treg) or NFAT1 KO (KO Treg) mice were co-cultured with CD4^+^CD25^−^ WT Te cells in different ratio of Te:Treg cells. (**d**) CD4^+^CD25^−^ effector T cells from WT (Te) and NFAT1 KO mice (KO Te) were co-cultured with CD4^+^CD25^+^ Treg from WT healthy mice. Percentage of suppression was measured by [H^3^]-thymidine incorporation assay. *n* = 5–10 mice per group. Experiments were repeated three times. Data are the average of three independent experiments; error bars indicate SD. *p < 0.05, **p < 0.005 and ***p < 0.001.

We next investigated whether the enhanced pathogenic properties of T cells in CHS-induced NFAT1 KO mice is a consequence of a defect in an intrinsic ability to induce tolerance in these mice. We first measured the basal expression levels of CHS effector molecules in CD4^+^ and CD8^+^ T cells isolated from disease free young healthy NFAT1 KO mice and found the levels to be comparable to WT cells (Chae *et al*., unpublished data). We then tested the effect of NFAT1 deficiency on the suppressor activity of Treg cells. NFAT1 KO mice possessed similar proportion of Foxp3^+^ Treg cells among their CD4^+^ T cells (Fig. 3a). However, in consistence to a recent report implicating NFAT’s role in the heritable maintenance of Foxp3, we observed reduced mean fluorescence intensity (MFI) of Foxp3 in NFAT1 KO cells (Fig. 3a). To compare the expression levels of Treg-associated molecules, we isolated and analyzed CD4^+^CD25^+^ T cells for these markers by qRT-PCR. CD4^+^CD25^+^ T cells from NFAT1 KO expressed significantly lower levels of Foxp3 and several Treg cell effector molecules, including IL10, CTLA4, Granzyme B/C, perforin and FasL (Fig. 3b). We also compared the suppressive function of CD4^+^CD25^+^ Treg cells between WT and NFAT1 KO by stimulating WT conventional CD4^+^CD25^−^ cells with anti-CD3 plus anti-CD28 mAbs in the presence of varying numbers of CD4^+^CD25^+^ cells. Strikingly, CD4^+^CD25^+^ cells from NFAT1 KO displayed decreased suppressive capability compared with WT CD4^+^CD25^+^ Treg cells (Fig. 3c). As a side note, conventional CD4^+^CD25^−^ T cells from NFAT1 KO mice displayed normal susceptibility to suppression mediated by WT CD4^+^CD25^+^ Treg cells (Fig. 3d). These data suggest that a functional defect in the Treg cell activity, presumably resulting from impaired maintenance of Foxp3 expression in the absence of NFAT1, might be one of possible causes for exacerbated CHS responses in NFAT1 KO mice.

### NFAT1 deficiency induces a defect in activation induced cell death of CD4^+^ and CD8^+^ T cells

**Figure 4:**
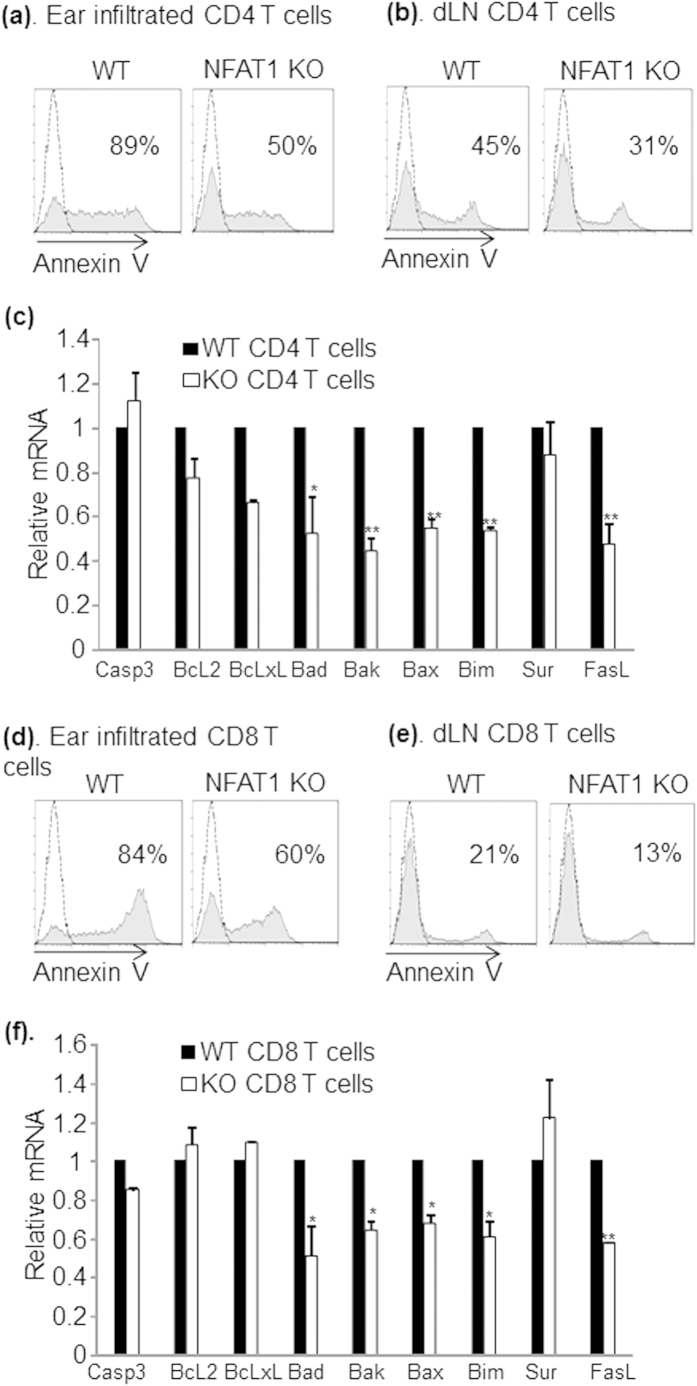
Defect in activation induced cell death program in NFAT1 KO mice. Sorted CD4^+^ or CD8^+^ T cells were obtained from the chronic stage of hypersensitivity in WT and NFAT1 KO mice. Apoptotic population (Annexin V^+^) of CD4^+^ or CD8^+^ T cells in infiltrated ear lesion (**a,d**) and draining LN (**b,e**) was measured by flow cytometry. Relative expression of apoptosis related molecules was measured by qRT-PCR in CD4^+^ (**c**) or CD8^+^ (**f**) T cells. Data are the average of three independent experiments; error bars indicate SD. *p < 0.05, **p < 0.005 and ***p < 0.001.

Among the diverse T cell tolerance programs, AICD is a key regulatory mechanism to prevent hyper-activation of immune system and a defect in this process can lead to hyper-immune disorders including skin inflammation^29^,^30^. We investigated the possibility that exacerbated pathogenesis of CHS in NFAT1 KO mice is caused by a defect in AICD of hapten reactive pathogenic T cells. To this end we measured the proportion of apoptotic cells among the CD4^+^ and CD8^+^ T cells isolated from inflamed tissues and draining lymph nodes (cervical and auxiliary lymph nodes) of WT and NFAT1 KO mice 4 weeks after induction of chronic CHS. Despite producing increased amounts of pro-inflammatory cytokines (Fig. 2d,f), CD4^+^ (Fig. 4a,b) and CD8^+^ (Fig. 4d,e) T cells isolated from NFAT1 KO mice possessed significantly reduced populations of Annexin V^+^ cells in comparison to WT mice. Furthermore, in consistence with this notion, both CD4^+^ and CD8^+^ T cells of NFAT1 KO mice expressed significantly lower levels of pro-apoptotic Bcl-2/BH3 family molecules^31^ including Bad, Bak, Bax, Bim as well as FasL (Fig. 4c,f). These data suggest that a defect in AICD program in NFAT1 KO mice may contribute to accumulation of antigen-activated pathogenic T cells, resulting in aggravated CHS pathogenesis.

### NFAT1 positively regulates the expression of pro-apoptotic genes

**Figure 5:**
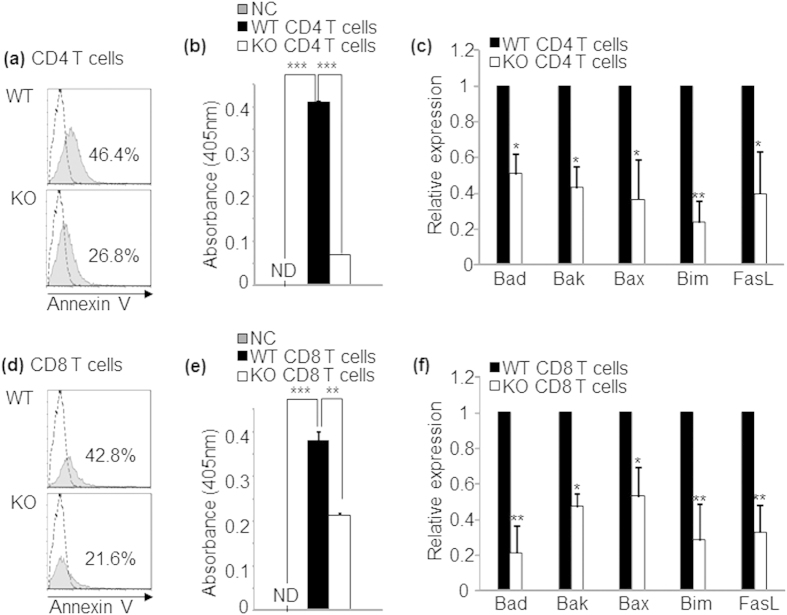
Down-regulation of apoptosis mediators in NFAT1 KO T cells. CD4^+^ or CD8^+^ T cells isolated from healthy WT or NFAT1 KO mice were stimulated with anti-CD3/anti-CD28 for 48 hrs and stimulation dependent T cell apoptosis was analyzed by Annexin-V staining in CD4^+^ (**a**) and CD8^+^ T cells (**d**) respectively. Dashed histogram is isotype control. Activity of active caspase-3 levels was measured in CD4^+^ (**b**) and CD8^+^ T cells (**e**). NC; normal healthy control without CHS induction. In the same condition, relative expression levels of pro-apoptotic molecules were quantified by qRT-PCR on sorted CD4^+^ (**C**) and CD8^+^ T cells (**f**). *n* = 5–10 mice per group. Experiments were repeated at least three times. Data are the average of three independent experiments; error bars indicate SD. *p < 0.05, **p < 0.005 ***p < 0.001, ND; no detection.

**Figure 6:**
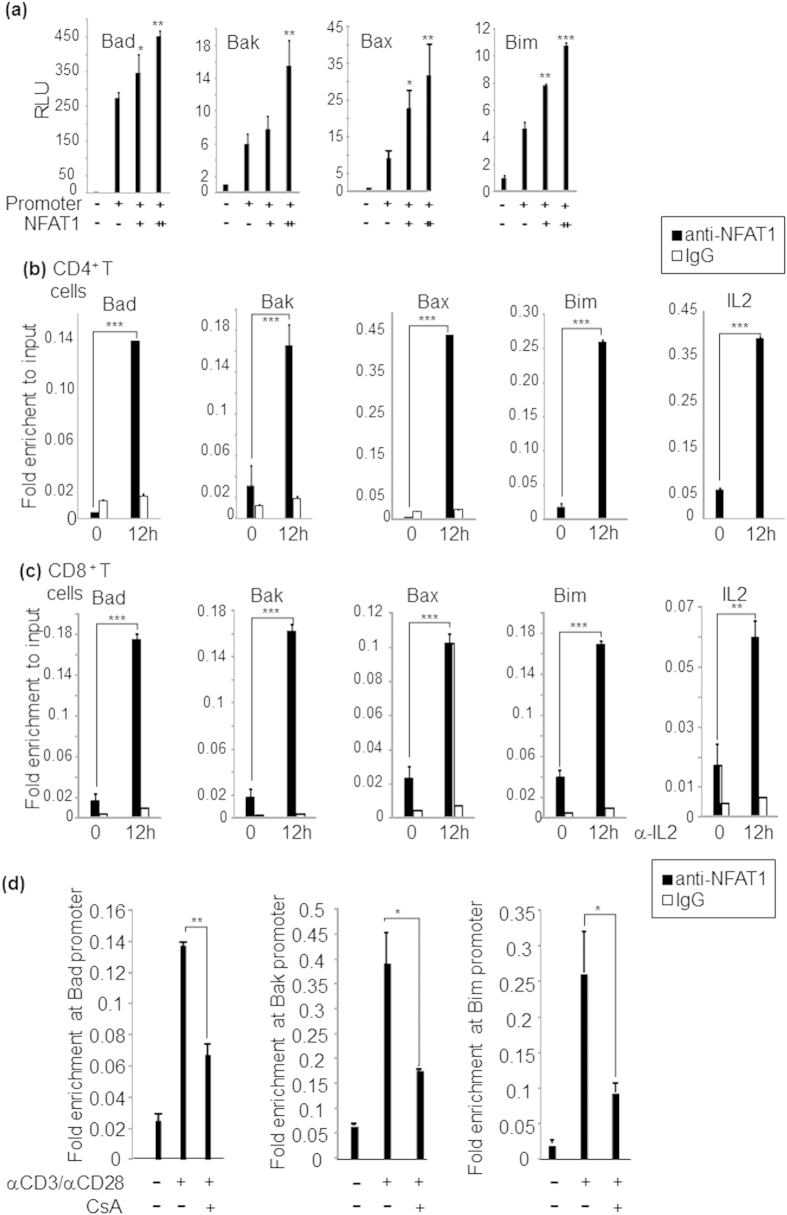
NFAT1 occupies promoter regions of genes encoding Bcl-2 family members and positively regulates their expression. (**a**) HEK293 cells were co-transfected with indicated luciferase reporter constructs with mock (pcDNA) or various concentrations of NFAT1 plasmid (−; 0 ng, +; 500 ng and ++; 1000 ng of NFAT1), and luciferase activities were measured. Normalized luciferase activity is expressed as a fold difference relative to the control activity. Endogenous NFAT1 binding to the promoter of each target gene in (**b**) CD4^+^ T cells and (**c**) CD8^+^ T cells was analyzed by qRT-PCR following ChIP assay in the absence or presence of α-CD3/α-CD28 stimulation for 12 hrs. The *Il2* promoter locus, a known target for NFAT1, is used as a positive control. (**d**) NFAT1 occupancy on the indicated loci of CD4 T cells was determined by ChIP analyses in the presence or absence of CsA under indicated conditions. Data are the average of three independent experiments; error bars indicate SD. *p < 0.05, **p < 0.005 and ***p < 0.001.

We next determined whether deficiency in NFAT1 renders T cells impaired in their ability to undergo AICD. To this end, T cells from young WT and NFAT1 KO mice free of skin inflammation were stimulated with α-CD3/α-CD28 and analyzed for emergence of Annexin V^+^ apoptotic cells by flow cytometry. In concert to the results obtained in the previous section, both CD4^+^ and CD8^+^ T cells obtained from NFAT1 KO mice displayed a significant reduction in the proportion of Annexin V^+^ cells with a lower caspase 3 activity compared to WT cells (Fig. 5a,b,d,e). Next, we assessed whether NFAT1 regulates the expression of pro-apoptotic BCL-2/BH3 family genes. Indeed, NFAT1 deficiency significantly reduced expression level of Bad, Bak, Bax, Bim and FasL in both CD4^+^ and CD8^+^ T cells (Fig. 5c,f). Since withdrawal of growth factors induces a mitochondria-dependent apoptosis pathway, we tested the effect of IL2 depletion on AICD progression. Indeed, blockade of IL2 by treating IL2 blocking antibody significantly increased apoptotic population in WT CD4^+^ T cells but not in NFAT1 KO cells (Supplementary Fig. S2). These data indicate that NFAT1 could turn on the expression of pro-apoptotic BCL-2/BH3 family genes for the induction of AICD triggered by growth factor starvation and/or antigen stimulation. In addition, Promoters of Bad, Bak, Bax and Bim are highly conserved (>70%) between mouse and human by TRANSFAC analysis^32^ (Supplementary Fig. S3). To define the role of NFAT1 as a transactivator of the genes encoding these proteins, luciferase reporter analysis was performed. Target promoter-driven luciferase reporter plasmids were co-transfected with different doses of NFAT1 expression plasmid and alteration in promoter activities was measured. Indeed, co-expression of NFAT1 significantly enhanced the promoter activities in a dose-dependent manner (Fig. 6a). On the other hand, mutation of NFAT1 binding sites in Bak promoter significantly decreased stimulation dependent promoter activity (Supplementary Fig. S4A,B). Next, we tested the physical association of NFAT1 with the promoter of target genes by performing chromatin immunoprecipitation (ChIP) assay. NFAT1 binding to *Il2* promoter regions was used as a positive control^33^. Binding of NFAT1 to the target promoters was significantly enriched upon α-CD3/α-CD28 stimulation of both CD4^+^ (Fig. 6b) and CD8^+^ T cells (Fig. 6c) isolated from WT mice. Consistent to the fact that nuclear entry of NFAT1 is dependent on canonical Ca^2+^/calcineurin pathway, the occupancy of NFAT1 on these loci was significantly reduced in the presence of the calcineurin inhibitor cyclosporine A (CsA) (Fig. 6d). Collectively, these results suggest that NFAT1 acts as a transcriptional activator to up-regulate expression of pro-apoptotic BCL-2/BH3 molecules in both CD4^+^ and CD8^+^ T cells by binding to their promoters.

### NFAT1 regulates activation induced cell death of CD4^+^ and CD8^+^ T cells

**Figure 7:**
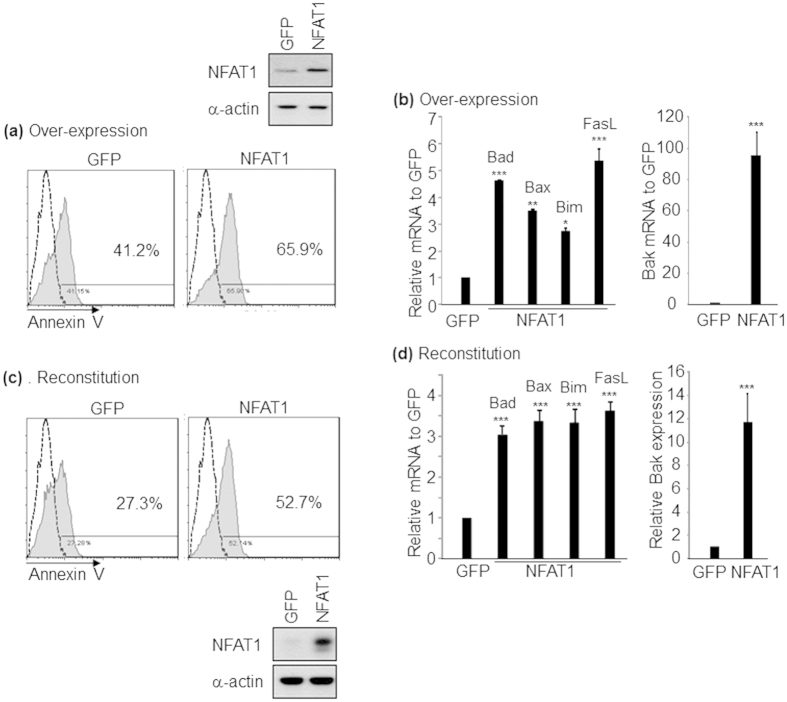
Reconstitution of NFAT1 restores activation induced cell death. (**a**) WT or (**C**) NFAT1 deficient CD4^+^ T cells were transfected with NFAT1 or control plasmid, and apoptotic population upon α-CD3/α-CD28 stimulation was compared by Annexin-V staining. Dashed histogram is isotype control. Expression level of apoptosis related molecules in over-expression (**b**) or reconstitution condition (**d**) was measured by qRT-PCR. Data are the average of three independent experiments; error bars indicate SD. *p < 0.05, **p < 0.005 and ***p < 0.001.

We next tested the effect of over-expressing NFAT1 on the apoptosis program. Wild type CD4^+^ T cells were transfected with either mock (GFP) or NFAT1 expressing plasmids, stimulated with α-CD3/α-CD28, and then emergence of apoptotic cells analyzed by flow cytometry. Over-expression of NFAT1 significantly enhanced generation of apoptotic cells (GFP; 41.2% vs. NFAT1; 65.9%) (Fig. 7a). NFAT1 over-expression also significantly up-regulated NFAT1 target genes such as FasL and pro-apoptotic Bcl2 family genes (Bad, Bak, Bax and Bim) (Fig. 7b). We also tested whether reconstitution of NFAT1 into NFAT1 KO CD4^+^ T cells could restore the apoptosis program. Indeed, reconstitution of NFAT1 sufficiently restored the Annexin V^+^ apoptotic population (Fig. 7c) by increasing the expression levels of pro-apoptotic molecules (Fig. 7d). Collectively, these results indicate that NFAT1 regulates AICD pathways through enhancing the expression levels of key apoptosis mediators.

### NFAT1 regulates activation induced cell death *in vivo*

**Figure 8:**
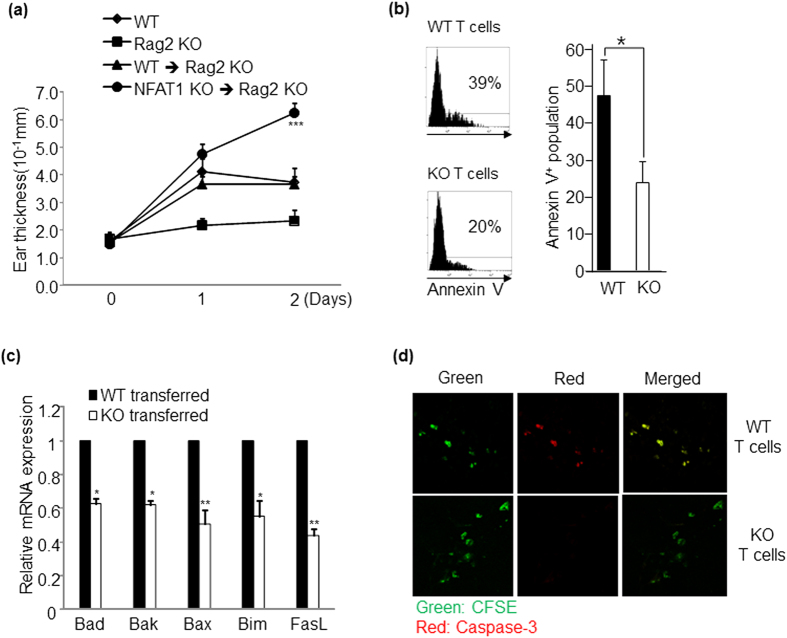
NFAT1 KO T cells are more resistant to activation induced cell death *in vivo*. CD4^+^ and CD8^+^ T cells isolated from WT or NFAT1 KO mice under chronic stage of contact hypersensitivity were labeled with CFSE and adoptively transferred to recipient Rag2^−/−^ mice that were challenged with DNCB on the ear. (**a**) Ear thickness was monitored at indicated time points. After two times challenging of DNCB, apoptotic population (Annexin-V^+^/CFSE^+^) (**b**) or expression level of apoptosis related molecules (**c**) in splenocytes isolated from the Rag2^−/−^ recipient mice were measured. (**d**) Apoptotic population of transferred T cells in inflamed tissues was analyzed by staining with active-Caspase-3 (Red) and CFSE (Green). Data shown are the representative among three independent experiments; error bars indicate SD. *p < 0.05, **p < 0.005 and ***p < 0.001.

We further tested whether NFAT1 deficiency also affects the activation induced T cell death *in vivo* by performing adoptive transfer experiment. CD4^+^ and CD8^+^ T cells were purified from the draining lymph nodes of WT or NFAT1 KO mice at the chronic stage of CHS. Cells were labeled with CFSE and then adoptively transferred to Rag2 KO mice that were pre-challenged with DNCB on ear 7 days before transferring. After transferring of T cells, recipient mice were re-challenged with DNCB on ear two times, and disease severity and T cell apoptosis were analyzed on ear. Recipients mice transferred with NFAT1 KO T cells showed significantly increased ear thickness (Fig. 8a). They also showed much less Annexin V^+^ signal (Fig. 8b) and lower levels of Bad, Bax, Bak, Bim and FasL (Fig. 8c). We also measured active Caspase-3^+^ apoptotic cells in the inflamed tissues of recipient mice. T cells transferred from WT mice expressed much higher level of active Caspase-3 (upper panel in Fig. 8d) than NFAT1 KO mice (lower panel in Fig. 8d). Collectively, these data suggest that NFAT1 regulates activation induced T cell death *in vivo*.

## Discussion

Allergic contact hypersensitivity is caused by a defect in down-regulation of inflammation associated tissue damage. In this study, we demonstrated that transcription factor NFAT1 plays a key role in the development and progression of skin inflammation. NFAT1 KO mice spontaneously developed a mild skin inflammation in old age, and were more susceptible to induction of experimental CHS. Enhanced CHS susceptibility was closely related with increased Th1/Th17 type responses as well as higher levels of CTL associated molecules. NFAT1 deficiency induced functional defect in Treg cells, and down-regulated a set of apoptosis associated genes such as FasL and pro-apoptotic Bcl-2/BH3 family molecules. Enhanced CHS symptoms in Rag2^−/−^ mice adoptively transferred with NFAT1 deficient CD4^+^/CD8^+^ T cells further confirmed the functional importance of NFAT1 in induction of activation induced T cell death *in vivo*.

Maintenance of immune homeostasis is pivotal process to keep our body in healthy condition. Removal of pathogens without inducing chronic inflammation mediated by activated T and B cells is critical to avoid hyper-immune disorders^30^. Immune tolerance is mediated in diverse ways^34^. Among them, one of the most effective way of turning down the hyper-activated or auto-reactive T cell responses is mediated by activation induced cell death (AICD) or apoptotic cell death^30^,^34^. AICD is an elaborately controlled program and various molecules are involved in this process. This program includes two major pathways, such as death receptor mediated signaling and Bcl-2 family mediated apoptotic signaling pathways^30^. Upon TCR stimulation, expression level of Fas ligand (FasL or CD95L) in T cells is rapidly up-regulated^35^, and NFAT1 activates FasL expression by directly binding on the promoter. In addition, NFAT1 induces Egr3 gene, which subsequently enhances FasL expression^19^. However, Fas/FasL pathway may play a minor role in inducing activation induced T cell death in response to conventional foreign antigen. Rather Fas/FasL pathway mainly controls the death of self-reactive T cells in peripheral immune system^36^,^37^. We also found that blockage of Fas/FasL interaction by treatment of blocking antibody (MFL4)^38^ only partially inhibits TCR stimulation-induced T cell death *in vitro* (Supplementary Fig. S5), suggesting an involvement of other major mechanisms to induce AICD in T cells. Indeed, accumulated data indicate a central role of Bcl-2 protein in AICD. This pathway is cooperatively regulated by various pro- (Bad, Bak, Bax and Bim)^39^,^40^,^41^ and anti-apoptotic (Bcl-2 and Bcl-xL)^42^,^43^ members of Bcl-2 protein family. However, it is still unclear how Bcl-2 family genes are regulated, and which kinds of molecules are involved in regulation of these genes at the transcription level. Interestingly, we found that expression of various pro-apoptotic genes including Bad, Bak, Bax and Bim was induced by TCR stimulation in both CD4^+^ and CD8^+^ T cells in a calcineurin dependent manner (Supplementary Fig. S5E). These findings indicate a possible involvement of NFAT1 in pro-apoptotic Bcl-2/BH3 family gene mediated apoptotic processes. NFAT1 positively regulates the Bcl-2/BH3 family genes by binding to the promoters of each target regions (Fig. 6). Reconstitution of NFAT1 expression in NFAT1 KO T cells restored T cell apoptosis by increasing the level of pro-apoptotic Bcl-2 family genes (Fig. 7). Based on our findings, we suggest that NFAT1 may act as a key molecular switch to turn on the program of activation induced cell death by regulating both death receptor (FasL)-mediated and pro-apoptotic Bcl-2/BH3 family-mediated apoptotic pathways.

NFAT cooperates with various transcription factors to synergistically regulate the expression of its down-stream target genes^28^. Among them, AP1 is a well-known binding partner of NFAT that forms a ternary NFAT:AP1 complex on DNA to regulate numerous genes involved in various cellular processes^44^. We tested the possible involvement of AP1 for the NFAT1 dependent activation of pro-apoptotic Bcl-2/BH3 family genes by performing promoter reporter assay. However, co-expression of NFAT1 with AP1 failed to synergistically activate Bak promoter, and mutation of NFAT binding site reduced NFAT1-drived promoter activity (Supplementary Fig. S4A,B). Moreover, over-expression of NFAT1 mutant protein that is unable to interact with AP-1^45^ also increased promoter activity of these genes with the comparable efficiency of WT NFAT1 (data not shown). Bioinformatic analyses of the promoter regions of BH-3 family genes also showed that NFAT binding sites are not co-localized with AP1 site, while binding sites for other co-factors such as NFκB, ERG1/2 and Ets1 are closely clustered (Supplementary Fig. S3). These studies suggest that NFAT1 may positively regulate pro-apoptotic Bcl-2/BH3 family genes in an AP1 independent manner for the induction of AICD in the case of T cell exhaustion^46^ or TNFα gene expression^44^.

Among five different NFAT families, NFAT1, NFAT2 and NFAT4 are mainly expressed in T lymphocytes and share DNA binding specificity and binding partners. Many of NFAT target genes are redundantly regulated by combination of these NFAT proteins conferring functional redundancy^28^. In this study, we also observed a partial, rather than, complete defect of AICD in NFAT1 KO T cells. We tested the effects of different NFAT proteins on the expression levels of pro-apoptotic Bcl-2/BH3 family genes. Among the tested NFAT proteins (NFAT1, NFAT2 and NFAT4), NFAT1 predominantly increased the expression levels of Bad, Bak, Bax and Bim upon TCR stimulation in WT CD4^+^ T cells (Supplementary Fig. S6). This result is consistent with the phenotype of NFAT1^−/−^ NFAT4^−/−^ mice that show more profound lymphoproliferative disorder^47^ than a NFAT1 KO mice, suggesting a certain degree of redundancy but unique and combinatory effects of NFAT1 together with each NFATs.

A defect in AICD among effector T cells appears to be a key phenotypic consequence in the absence of NFAT1 that leads to enhanced CHS. However, a relatively minor role of Foxp3^+^ regulatory T cells cannot be ruled out. While NFAT-Smad3 complex upon TGFß signaling is known to be important for peripheral Treg generation, NFAT2, and not NFAT1 has been shown to be the key player in this scenario. Therefore it seems unlikely that NFAT1 deficiency leads to a reduced number of pTreg cells in these mice. However we did observe a significant reduction in the steady state MFI of Foxp3 expression (Fig. 3) in NFAT1 deficient Treg cells. This is in concert to a recent finding that NFAT1, and to some extent NFAT2, upon TCR stimulation associates with the CNS2 region of *Foxp3*, resulting in the optimal maintenance of Foxp3 under inflammatory conditions^27^. It seems likely that CHS driven inflammation does result in reduced maintenance of Foxp3 expression in NFAT1 deficient Treg cells in a CNS2 dependent manner, resulting in compromised suppressive activity under steady state condition. Lastly, albeit in a redundant way, a direct involvement of NFAT1 in mediating suppressive property of Treg cells cannot be ruled out. While NFAT1 associates with Foxp3 dimers and play an important role in *Il2* gene repression^25^,^48^, Treg cells derived from NFAT1^−/−^NFAT4^−/−^ double knock-out cells appear to be functional^49^. On the other hand restricting nuclear translocation of essentially all NFAT proteins by Treg specific deletion of Cnb1 results in loss of function and altered gene expression profile in Cnb1 deficient Treg cells^26^. Taken together these findings suggest some level of redundancy among NFAT function in Treg mediated suppressive activity. It seems possible, at an older age or under experimental CHS conditions a compromised suppressive capacity of NFAT1 deficient Treg cells is manifested more than at younger, unchallenged scenario. In this regard, while there was no significant differences among Helios^+^ nTreg cells, we observed increased level of CCR6^+^ in NFAT1 KO mice (Supplementary Fig. S7), suggesting possible roles of NFAT1 for the modulation of skin related homing function in Treg cells, a role of NFAT1 that is consistent with other cell types such as keratinocyte^50^ or DCs (Chae *et al*., unpublished data).

In summary, we found that NFAT1 plays a key role in pathogenesis of contact hypersensitivity-mediated skin inflammation by regulating AICD program. NFAT1 positively regulates pro-apoptotic Bcl-2/BH3 family genes (Bad, Bak, Bax and Bim) and death receptor mediated pathway (FasL). Conclusively, our results suggest that NFAT1 may serve as a molecular switch to turn on AICD program for the maintenance of peripheral tolerance in both CD4^+^ and CD8^+^ T cells.

## Materials and Methods

### Mice

C57BL/6 mice (6 ~ 8 weeks) were purchased from SLC Inc. (Hamamatsu, Japan) and NFAT1^−/−^ KO mice were kindly provided by Dr. Anjana Rao (La Jolla Institute for Allergy & Immunology, CA, USA). All mice were maintained under specific pathogen-free conditions in the animal facility of the Gwangju Institute of Science and Technology (GIST). All experimental procedures were performed in accordance with National Institutes of Health (NIH) Guidelines for the care and use of laboratory animals, and were approved by Animal Care and Ethics Committees of GIST. Animals were maintained in accordance with the National Animal Welfare Law of Korea.

### Murine contact hypersensitivity (CHS) disease model

To induce acute type of contact hypersensitivity, mice were sensitized by topical application of 100 μl of 4% 2,4-Dinitrochlorobenzene (DNCB) (Sigma Aldrich, St Louis, MO, USA) dissolved in acetone/olive oil (1:3, v/v) solution on both sides of ears at Day 0. After 3 days of sensitization, mice were challenged by topical application of 20 μl of 2% DNCB in acetone/olive oil (1:3; v/v). After 6, 12, 24, 36 and 48 hours of challenge, ear thickness and clinical symptoms were monitored. For induction of chronic contact hyper-sensitivity, 7 days after sensitization, mice were repeatedly challenged with 20 μl of 2% DNCB twice a week at 3 days interval for 4 weeks. After 12 hrs of every challenge, ear thickness and clinical symptoms were monitored.

### Histology

Clinical condition and symptoms of each mice were evaluated by histological analysis. H&E staining was performed with a minor modification of previously described method^51^. Briefly, ear tissues were collected and fixed in 4% formaldehyde for 12 hrs. After fixation, tissues were embeded in paraffin blocks, sectioned at 3 μm thickness and stained with Hematoxylin (Sigma Aldrich, St Louis, MO, USA) and Eosin (Sigma Aldrich, St Louis, MO, USA).

### Isolation of CD4^+^ T cells, CD8^+^ T cells, CD4^+^CD25^+^ T cells and CD4^+^CD25^−^ T cells

For isolation of primary immune cells, spleens or local draining lymph nodes were used according to each experimental purpose. For the isolation of specific cell types, splenic or lymph node total cells were incubated with CD4^+^ or CD8^+^microbeads (Miltenyi Biotech, Germany) following manufacturer’s protocol. For isolation of CD4^+^CD25^−^ effector T cells and CD4^+^CD25^+^ regulatory T cells (Treg), CD4^+^ T cells were isolated by mouse CD4 Dynabeads (Invitrogen, NY, USA; Cat No. 114.45) and DETACHaBEAD (Dynal; Cat No.124.06D). For the isolation of CD4^+^CD25^+^ T cells, isolated CD4^+^ T cells were labeled with biotin-conjugated rat anti-mouse CD25 (BD Pharmingen; Cat No. 553069) and incubated with streptavidin microbeads (Miltenyi Biotech, Germany; Cat No. 130-048-101) in a labeling buffer (PBS pH7.2, 2 mM EDTA). For the isolation of the cells from inflamed ear tissues, tissues were digested with 0.5 mg/ml of type V collagenase (Sigma Aldrich, St Louis, MO, USA) and then washed 5 times with PBS containing 10% FBS and 1X PS cocktail as described previously^51^. To isolate tissue infiltrated CD4^+^ or CD8^+^ T cells, total cells obtained after tissue digestion were further incubated with CD4^+^ or CD8^+^ magnetic beads followed by previously described method^51^.

### Cell culture

HEK293 cells were maintained in DMEM (Welgene, Daegu, Korea) and mouse primary CD4^+^ T and CD8^+^ T cells were cultured in T cell medium containing RPMI (Welgene, Daegu, Korea) supplemented with 10% fetal bovine serum (HyClone, USA), 3 mM L-glutamine (Sigma Aldrich, St Louis, MO, USA), 10 mM HEPES (Sigma Aldrich, St Louis, MO, USA), 100 U/ml penicillin , streptomycin (Sigma Aldrich, St Louis, MO, USA), and 0.05 mM 2-beta-mercaptoethanol (Sigma Aldrich, St Louis, MO, USA). For proper activation of primary cells, T cells were activated with plate-bound anti-CD3 and soluble anti-CD28 (1 μg/ml) (BD Bioscience). To inhibit nuclear translocation of NFAT, cells were pretreated with 1 μM Cyclosporin A (Calbiotech, CA, USA) for 12 hrs before stimulation with anti-CD3 and anti-CD28.

### RNA isolation, cDNA synthesis and quantitative RT-PCR

Total RNA was isolated from each sample and cDNA was prepared by reverse transcription using reverse transcriptase (Promega, Madison, WI, USA) and oligo(dT) primers as previously decribed^51^. The synthesized cDNAs were amplified by quantitative-real-time PCR (qRT-PCR) and standard PCR.

### Analysis of Caspase 3 activity

Caspase 3 activity was measured with CaspACE assay system (Promega, Madison, WI, USA, Madison, WI, USA) according to the manufacturer’s instructions. Briefly, total proteins were extracted in cell lysis buffer (Promega, Madison, WI, USA) from stimulated or non-stimulated cells, and protein concentration of each sample was determined by Bradford assay (Bio-Rad). To detect caspase 3 activity in each sample, same amount of protein was incubated with 2 μl of DEVD-pNA substrate for 4 hrs at 37 °C. After incubation, caspase 3 activity of each sample was measured by spectrophotometer at 405 nM.

### Flow cytometric analysis

To detect the types of immune cells infiltrated into tissue, anti-CD11b-PE (eBioscience; M1/70) and anti-Gr1-PE (eBioscience; RB6-8C5) were used for labeling. To determine the levels of intracellular cytokines, stimulated cells were treated with Brefeldin A (eBioscience) for 12 hours, harvested and permeabilized with intra-cellular staining buffer containing 0.1% saponin for 20 mins. After membrane permeabilization, cells were stained with proper antibodies (anti-IFNγ; XMG1; BD bioscience, anti-IL17A-PE; eBio17B7; eBioscience and anti-TNFα-FITC; MP6-XT22; eBioscience) and analyzed by flow cytometry. To analyze CD4^+^Foxp3^+^ regulatory T cells, isolated CD4^+^ T cells were fixed with Fixation/Permeabilization buffer (eBioscience) and stained with anti-Foxp3-PE (FJK-16s; eBioscience) in permeabilization buffer (eBioscience). To check apoptotic population, cells (1 × 10^6^) were washed with PBS and resuspended in 1 ml of 1X Annexin V binding buffer (BD bioscience). After incubating for 15 min with 5 μl of Annexin V-PE (BD bioscience), at 25 °C in the dark, 400 μl of 1× binding buffer was added to each tube and immediately analyzed by FACS. In an adoptive experiment, transferred T cells labeled with CFSE (Invitrogen, NY, USA) as shown in previous study^51^ from spleen were stained with Annexin V-PE and CFSE^+^/Annexin-V^+^ population was analyzed. Cells stained with isotype matched normal IgGs used as control and showed less than 0.2% positive population.

### Luciferase reporter assay

Promoter activities were measured by a method previously described^52^. Briefly, each promoter construct was transfected into HEK293 cells without or with several doses of NFAT1 expression plasmid. After 24 hrs, cells were stimulated with PMA (Calbiotech, CA, USA, CA, USA) and ionomycin (Calbiotech, CA, USA) for 4 hrs, collected and lysed in passive lysis buffer (Promega, Madison, WI, USA). Luciferase activity measured by dual luciferase assay system (Promega, Madison, WI, USA) was expressed relative to expression of the co-transfected Renilla luciferase promoter (hRluc; Promega, Madison, WI, USA) as control for transfection efficiency. Human Bak reporter construct was kindly gifted by Dr. Yong-Sung Juhnn, Seoul National University College of Medicine, Korea. Murine Bad, Bax and Bim promoters were cloned into PGL3 plasmid. The Bak reporter plasmid was used as the template for amplification reactions with the QuikChange II Site-Directed Mutagenesis kit (Agilent technologies, Santa Clara, CA, USA) according to the manufacturer’s protocol. Primers were designed to introduce mutations into one NFAT binding site: 5′-CTG TTA GCC GCA AAC AAT CTA TGA GAG AGC CTA AGA TAT ACT CTC CCA CTT AGG-3′ and 5′-CCT AAG TGG AGA GTA TAT CTT AGG CTC TCA TAG ATT GTT TGC GGC TAA CAG-3′.

### *In vitro* proliferation assay

To test hapten specific proliferation of CD4^+^ or CD8^+^ T cells, mitomycin treated splenocytes from WT mice were incubated with 10 mM of 2,4-Dinitrobenzene sulphonic acid dihydrate (Sigma Aldrich, St Louis, MO, USA) for 10 mins, washed 3 times with cold PBS and co-cultured with CD4^+^ or CD8^+^ T cells at 1: 10 ratio in 200 μl of T cell medium for 56 ~ 72 hrs in flat-bottomed 96-well plates. After 56 ~ 72 h culture, 0.5 μCi of H^3^-thymidine (NEN) was added to each well and the cells were incubated for an additional 16 hrs. Cells were harvested and H^3^-thymidine uptake was measured by liquid scintillation counting.

### CD4^+^ T cell differentiation and activation of CD8^+^ T cell

CD4^+^ T cells were purified from the lymph nodes and spleen using magnetic beads (L3T4, Miltenyi, Germany). For Th differentiation, the cells (5 × 10^6^/ml) were stimulated with plate-bound anti-CD3(1 μg/ml) and soluble anti-CD28 (2 μg/ml) under Th1-skewing (10 ng/ml IL12 and 10 μg/ml anti-IL4)^53^ or Th17-skewing (10 μg/ml of anti-IL4, 10 μg of anti-IFNγ, 5 ng/ml of TGFβ, and 10 ng/ml of IL6) conditions for 5 days, and re-stimulated with anti-CD3 (1 μg/ml) and soluble anti-CD28 (1 μg/ml) for further analysis. To generate effector cytotoxic T cells, CD8^+^ T cells were stimulated with of anti-CD3 (3 μg/ml) and soluble anti-CD28 (1 μg/ml) for 3 days, harvested and washed with PBS. Cells were then cultured in 100 U/ml of IL2 containing T cells medium for further 4 days and re-stimulated with 1 μg/ml of anti-CD3/anti-CD28 for further experiments as previously described^54^.

### *In vitro* suppression assay

To compare the suppression capacity of WT and NFAT1 KO CD4^+^CD25^+^ regulatory T cells (Treg; suppressor cells), CD4^+^CD25^+^ T cells isolated from WT and NFAT1 KO mice were co-cultured with WT splenic CD4^+^CD25^−^ T cells (effector cells) in the presence of mitomycin treated splenocytes and soluble anti-CD3 (1 μg/ml) at indicated suppressor and effector cell ratios. To test a susceptibility to suppression mediated by CD4^+^CD25^+^ Treg cells isolated from WT and NFAT1 KO mice, CD4^+^CD25^−^ effector cells from WT or NFAT1 KO mice were cultured with WT CD4^+^CD25^+^ suppressor cells in the presence of mitomycin treated splenocytes and soluble anti-CD3 (1 μg/ml) at indicated suppressor and effector cell ratios. Each sample was triplicated and culture was maintained up to 72 hrs. After 56 ~ 72 h culture, 0.5 μCi of (H^3^)-thymidine (NEN) was added to each well, and cells were incubated for an additional 6 h to measure (H^3^)-thymidine uptake by liquid scintillation counting.

### Chromatin immunoprecipitation (ChIP) assay

ChIP assay was performed with minor modification as previously described^53^. Briefly, CD4^+^ T cells (2 ~ 3 × 10^7^/sample) were cross-linked with formaldehyde at a final concentration 1% for 10 mins at RT, lysed, and sonicated to shear DNA to have 1000 ~ 500 base pairs. DNA concentration was measured and 200 μg of total fragmented DNA was used for ChIP assay with NFAT1 antibodies (a mixture of 5 μg of each of N-terminal and C-terminal targeted antibody)^55^. Anti-rabbit IgG (Sigma Aldrich, St Louis, MO, USA) was used to define background binding. Relative binding of NFAT1 to the specific locus was detected by PCR method. For the quantitative analysis, data were presented as the amount of DNA recovered relative to the input control, and differences between chromatin preparations were normalized using qRT-PCR.

### Computational analysis

To compare protein binding in each promoter, DNA motif analysis was performed using the PROMO and JASPAR programs^56^. The results from the two programs were combined.

### Immunohistochemistry

Immunochemistry was performed with 10 μm cryosection or 3 μm of paraffin section from back skin tissues, as previously described^51^. Briefly, to analyze CFSE^+^ transferred T cells in inflamed tissue lesion, tissue sections were stained with propidium iodide (Invitrogen, NY, USA) as a counter-staining targeting nucleus. Degree of CFSE labeled T cells infiltrated into tissue was monitored by confocal microscopic observation. For staining of the active caspase-3, tissue sections were boiled with citrate buffer in microwave for 20 mins for antigen retrieving, blocked with 3% BSA to inhibit non-specific binding and stained overnight at 4 °C with rabbit anti-cleaved caspases-3 (Cell Signaling). For the visualization, tissues sections were stained with Alexa-594 conjugated rabbit-IgG and CFSE^+^ cleaved caspase-3^+^ population were observed with confocal microscope.

### Adoptive transfer experiment

For the active induction of allergic contact hypersensitivity by transferring hapten reactive T cells, CD4^+^ and CD8^+^ T cells were isolated from CHS induced WT or NFAT1 KO mice. Isolated T cells (3 × 10^7^; a mixture of 2 × 10^7^ and 1 × 10^7^ of CD4^+^ and CD8^+^ T cells, respectively) were stained with CFSE (Invitrogen, NY, USA) and intraveously trasferred into Rag2 KO mice. Same number of WT T cells were used as a control. After 12 hrs of T cell transferring, mice were sensitized with DNCB and challenged for additional 2 times with DNCB at 3 day interval for 1 week. During induction period, ear thickness and clinical symptoms at inflamed site were monitored.

### Statistical analysis

Data are the mean ± SD of at least three independent experiments, unless differently specified in the text. A Student’s t-test was used to calculate the statistical significance of the experimental data. The level of significance was set at <0.05 were considered significant. Single asterisks (*) indicate p < 0.05, double asterisks (**) indicate p < 0.005, and triple asterisks (***) indicate p < 0.001, respectively.

## Additional Information

**How to cite this article**: Kwon, H.-K. *et al*. Transcription factor NFAT1 controls allergic contact hypersensitivity through regulation of activation induced cell death program. *Sci. Rep*. **6**, 19453; doi: 10.1038/srep19453 (2016).

## Supplementary Material

Supplementary Information
