# Supplementary material for: Transcription factor NFAT1 controls allergic contact hypersensitivity through regulation of activation induced cell death program
Source: Sci Rep. 2016 Jan 18;6:19453. doi: 10.1038/srep19453 (PMC4726067; doi:10.1038/srep19453)
Supplement: Supplementary Information [file srep19453-s1.pdf]

# Transcription factor NFAT1 controls allergic contact hypersensitivity through regulation of activation induced cell death program

Ho-Keun Kwon[1,2,#], Gi-Cheon Kim[1,3,#], Ji Sun Hwang[3], Young Kim[4], Chang-Suk Chae[3] Jong Hee Nam[4] , Chang-Duk Jun[1], Dipayan Rudra[3], Charles D. Surh[3,5] and Sin-Hyeog Im[3,5*]

[1]School of Life Sciences, Gwangju Institute of Science and Technology (GIST), 261 Cheomdan-gwagiro, Buk-gu, Gwangju, Republic of Korea 500-712

[2]Current address: Department of Microbiology and Immunobiology, Harvard Medical School, Boston, Massachusetts, USA 02115

[3]Academy of Immunology and Microbiology (AIM), Institute for Basic Science (IBS), Pohang, 790-784, Republic of Korea

[4]Chonnam National University Medical School, Gwangju 501-749, Korea

[5]Division of Integrative Biosciences and Biotechnology (IBB), Pohang University of Science and Technology, Pohang, Republic of Korea 790-784

[#] Equally contributed to this work

***Address correspondence to:** Dr. Sin-Hyeog Im, Academy of Immunology and Microbiology (AIM), Institute for Basic Science (IBS), Pohang, Republic of Korea; Division of Integrative Biosciences and Biotechnology (IBB), Pohang University of Science and Technology, Pohang, Republic of Korea 790-784; Tel: 82-54-279-2356; FAX: 82-54-279-8768; Email: iimsh@postech.ac.kr

# Supplementary Material

## Supplementary Figure Legends

**Supplementary Figure 1. Up-regulation of pro-inflammatory cytokines in CD4$^+$ T cells from aged NFAT1 KO mice.**

CD4$^+$ T cells isolated from spleen and lymph node of aged NFAT1 KO or WT littermate mice were stimulated with anti-CD3/anti-CD28, and level of cytokine production of was analyzed by flow cytometery. Data are the representative among three independent experiments.

**Supplementary Figure 2. Effects of IL-2 depletion for ACID**

CD4$^+$ T cells from NFAT1 KO or WT littermate were stimulated with anti-CD3/anti-CD28 , then re-stimulated with plate-coated anti-CD3 in the presence or absence of anti-IL-2 antibody for 12 hrs. Apoptotic population was analyzed by flow cytometry. Data are the representative among three independent experiments.

**Supplementary Figure 3. Bioinformatic analysis of promoter regions of representative BCL-2/BH3 family genes**

(A) Bad (mouse), (B) Bax (mouse), (C) Bak (human) and (D) Bim (mouse) promoter sequences were analyzed by PROMO and/or JASPAC. Predicted binding sites for NFAT and its co-factors are highlighted in different color.

**Supplementary Figure 4. Activity of Bak promoter is mainly mediated by NFAT1**

HEK293 cells were co-transfected with (A) WT or (B) Mutated NFAT1 site containing Bak reporter construct or mock (pcDNA) plasmid and NFAT1 (500ng) or NFAT1 (500ng) plus AP1 (pc-JUN and c-fos) plasmids (500ng), and then luciferase activities were measured. Normalized luciferase activity is expressed as the fold difference relative to the control activity. Data are the average of three independent experiments; error bars indicate SD. *$p < 0.05$

**Supplementary Figure 5. Blocking Fas-FasL interaction partially rescues T cells from AICD**

Activation induced apoptosis was analyzed by Annexin V staining in (A) $CD4^+$ T cells or (C) $CD8^+$ T cells stimulated with α-CD3/α-CD28 in the absence or presence of CsA. (B) $CD4^+$ T cells or (D) $CD8^+$ T cells were stimulated for indicated time points in the absence or presence of FasL blocking antibody (MFL; 1mg/ml or 10mg/ml), and apoptotic population (Annexin $V^+$) was measured by flow cytometry. Dashed histogram is isotype control. (E) Effect of CsA treatment on the expression levels of apoptosis mediators were measured by qRT-PCR. Data are the representative among three independent experiments; error bars indicate SD. *p <0.05, **p <0.005 and ***p <0.001.

**Supplementary Figure 6. Differential contributions of NFAT family members on pro-apoptotic gene expression.** WT $CD4^+$ T cells were transfected with indicated NFATs or sham plasmid and the expression level of apoptosis related molecules were analyzed by qRT-PCR. (A) FasL, (B) Bad, (C) Bak, (D) Bax and (E) Bim. Data are the average of three independent experiments; error bars indicate SD. *p <0.05, **p <0.005 and ***p <0.001.

**Supplementary Figure 7. Analysis $CD4^+Foxp3^+$ Treg cell subtypes between NFAT1 KO or WT littermate.**

Comparison of (A) Helios, (B) CCR5 and CCR6 expression on $CD4^+CD25^+Foxp3^+$ cells. Splenic and lymph node cells of WT and NFAT1 KO mice were stained with antibodies directed against CD4, CD25, Foxp3, Helios (A) and CCR5 (B, left panel) or CCR6 (B, right panel) and analyzed by flow cytometry. The histogram plots represent cells gated on $CD4^+CD25^+Foxp3^+$ expression. One of three independent experiments with cells pooled from 2 to 3 animals are shown.

Supplementary Fig. 1

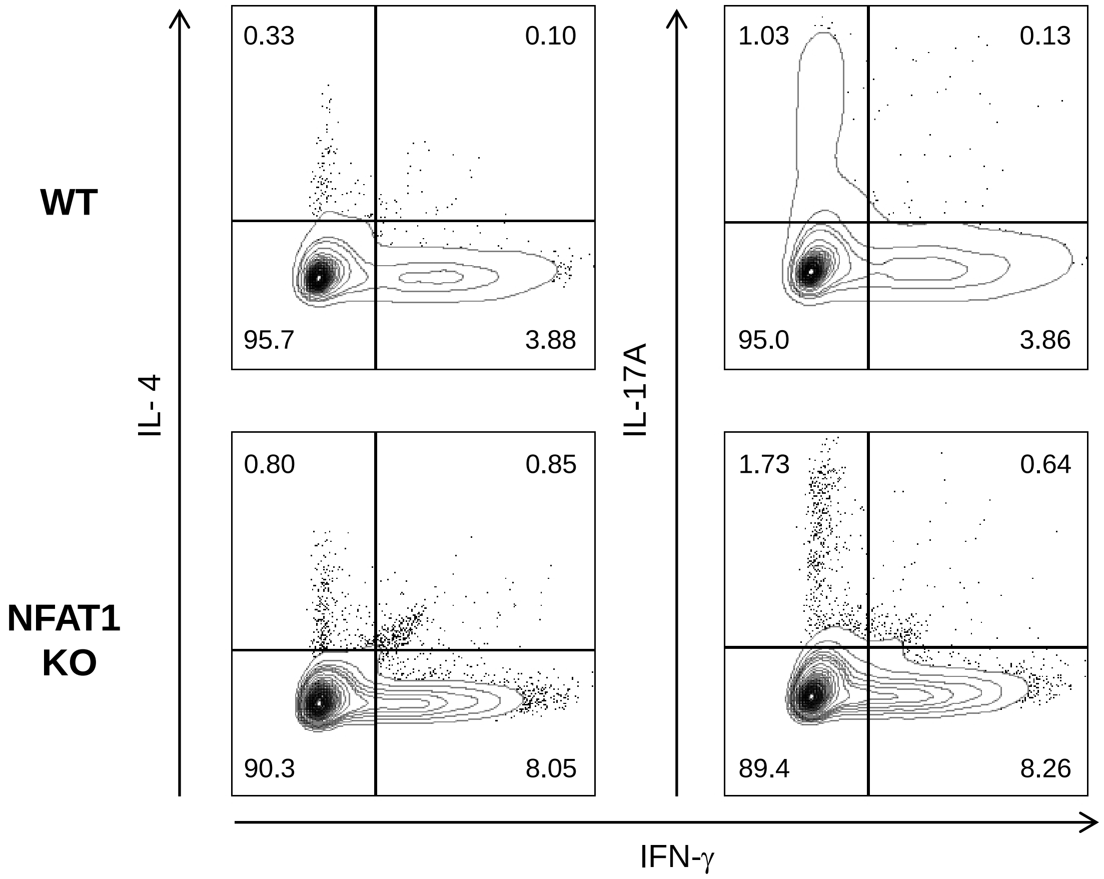

Supplementary Fig. 2

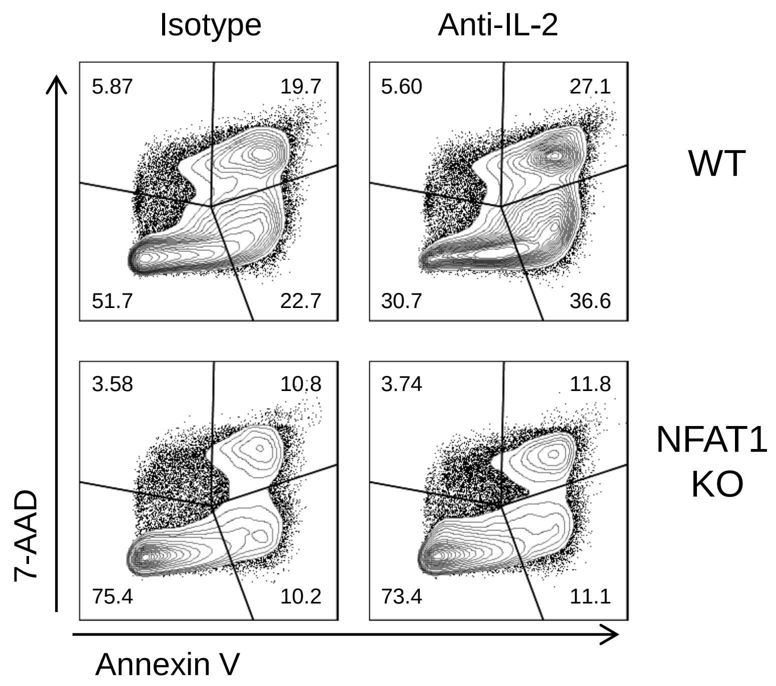

Supplementary Fig. 3

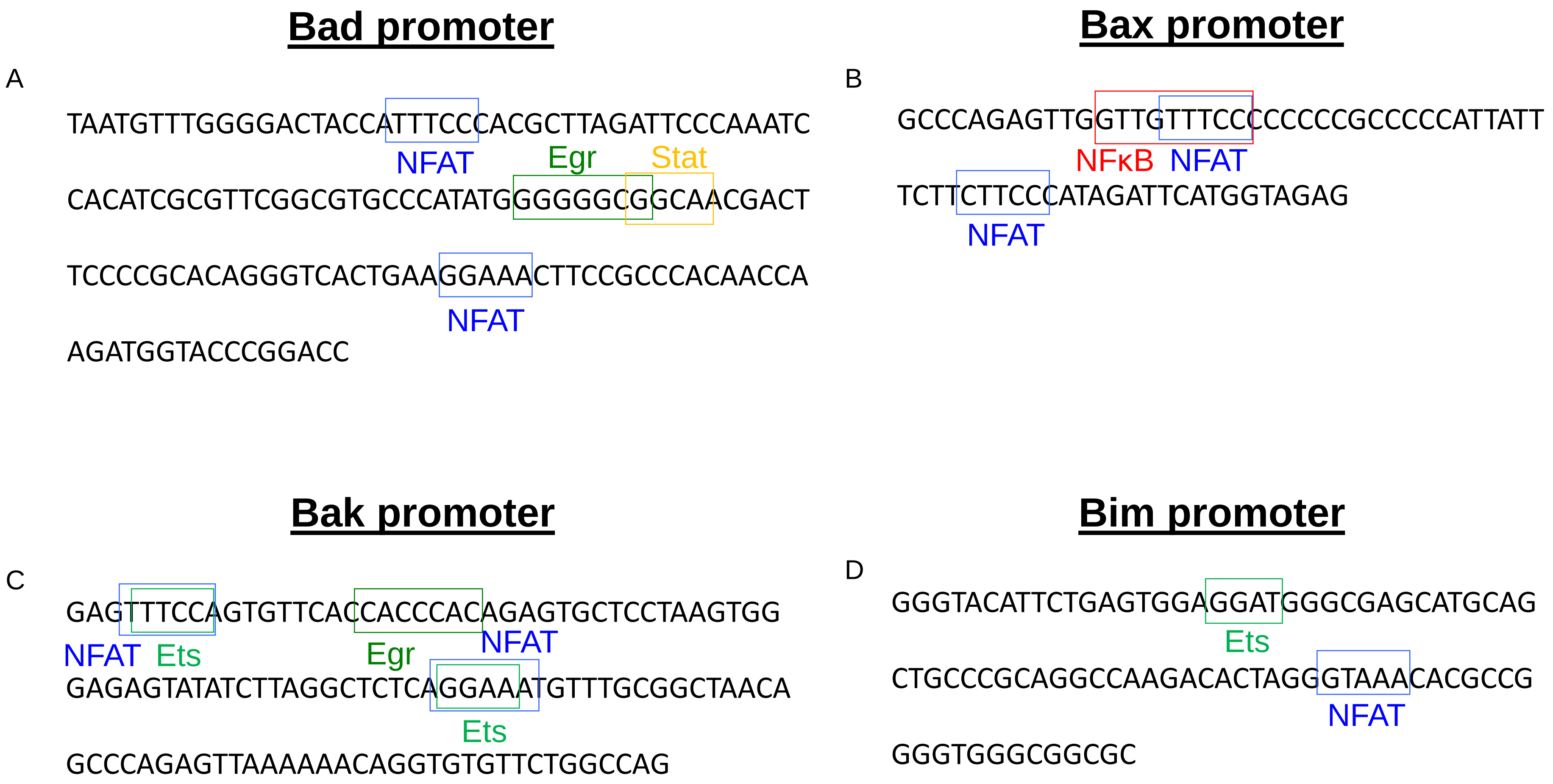

A.

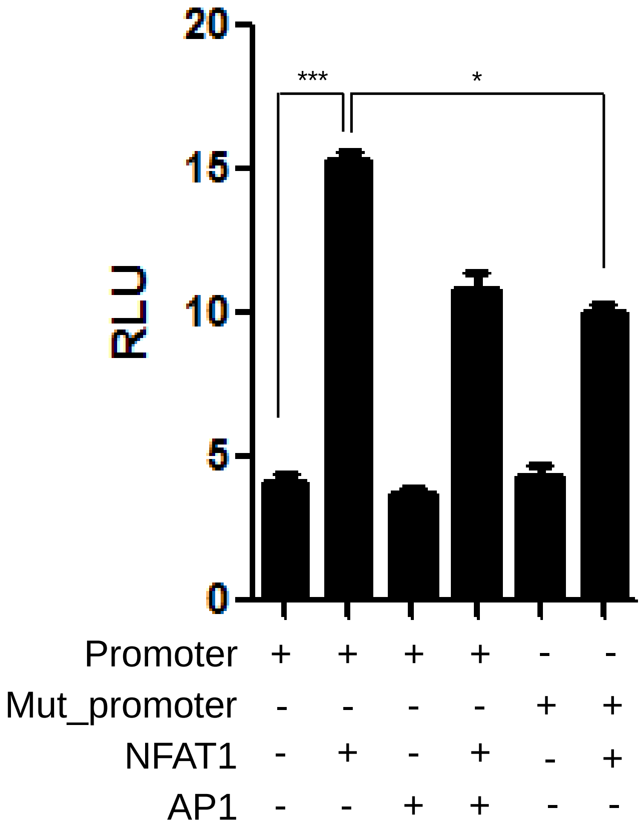


B. NFAT1 binding site and mutation sites in Bak promoter

WT: GAGTATATCTTAGGCTCTCA***GGAAA***TGTTTGCGGCTAACAGCCCA

Mutation: GAGTATATCTTAGGCTCTCA***TAGAT***TGTTTGCGGCTAACAGCCCA

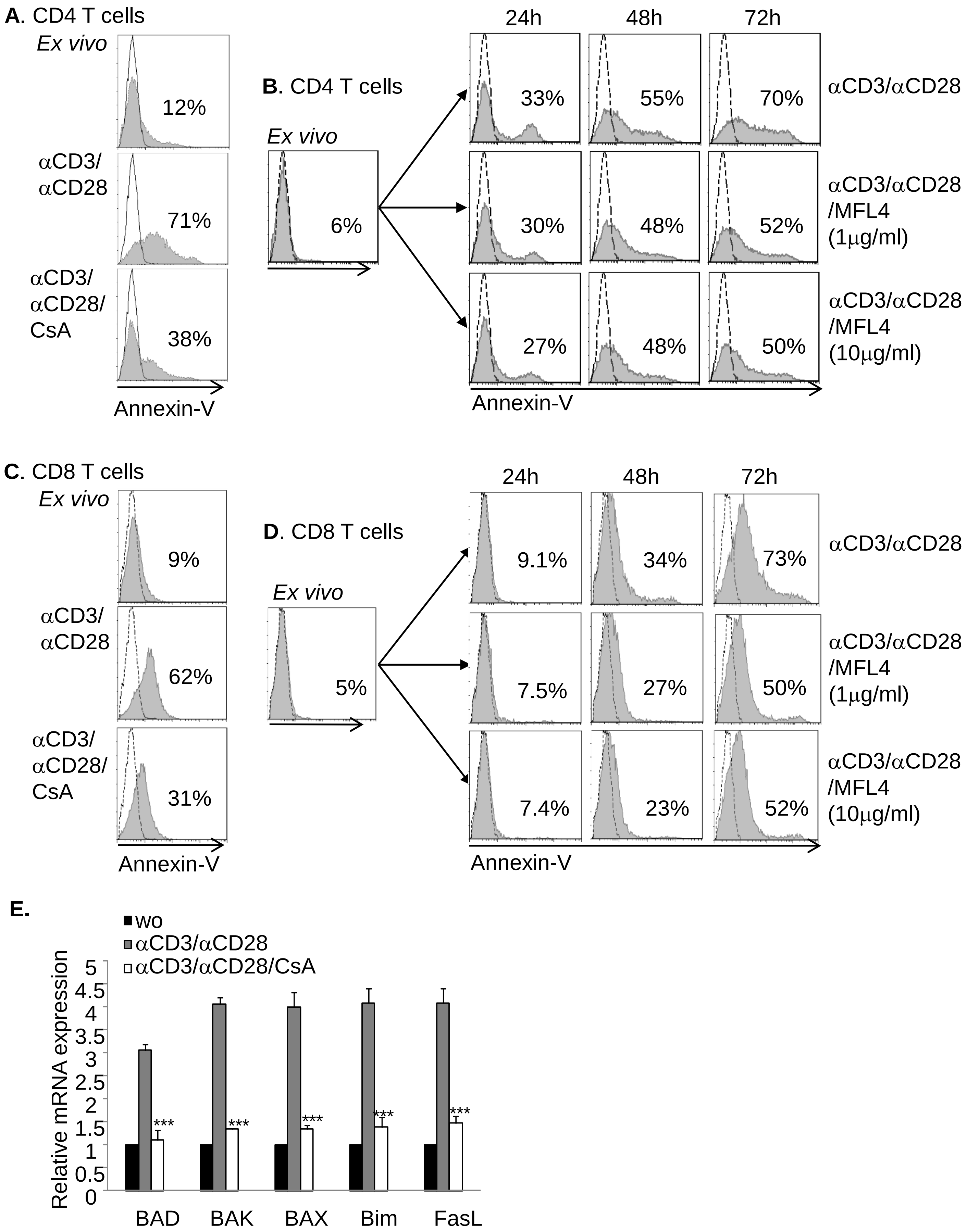

A. CD4 T cells
Ex vivo
12%
αCD3/αCD28
71%
αCD3/αCD28/CsA
38%
Annexin-V
B. CD4 T cells
Ex vivo
6%
24h
48h
72h
33%
55%
70%
αCD3/αCD28
30%
48%
52%
αCD3/αCD28/MFL4 (1μg/ml)
27%
48%
50%
αCD3/αCD28/MFL4 (10μg/ml)
Annexin-V
C. CD8 T cells
Ex vivo
9%
αCD3/αCD28
62%
αCD3/αCD28/CsA
31%
Annexin-V
D. CD8 T cells
Ex vivo
5%
24h
48h
72h
9.1%
34%
73%
αCD3/αCD28
7.5%
27%
50%
αCD3/αCD28/MFL4 (1μg/ml)
7.4%
23%
52%
αCD3/αCD28/MFL4 (10μg/ml)
Annexin-V
E.
wo
αCD3/αCD28
αCD3/αCD28/CsA
Relative mRNA expression
0
0.5
1
1.5
2
2.5
3
3.5
4
4.5
5
***
BAD
BAK
BAX
Bim
FasL

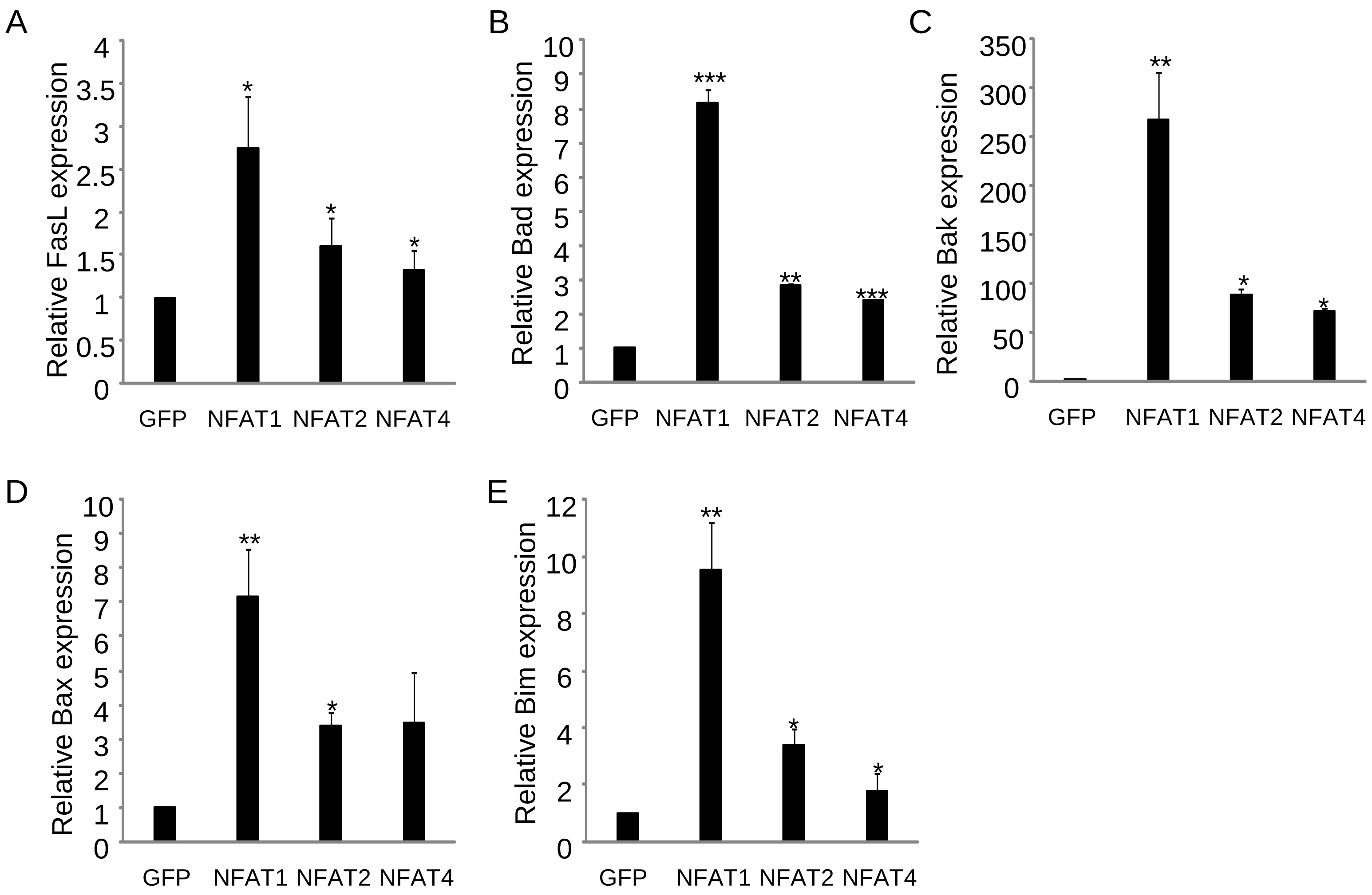
A
Relative FasL expression
4
3.5
3
2.5
2
1.5
1
0.5
0
GFP NFAT1 NFAT2 NFAT4
B
Relative Bad expression
10
9
8
7
6
5
4
3
2
1
0
GFP NFAT1 NFAT2 NFAT4
C
Relative Bak expression
350
300
250
200
150
100
50
0
GFP NFAT1 NFAT2 NFAT4
D
Relative Bax expression
10
9
8
7
6
5
4
3
2
1
0
GFP NFAT1 NFAT2 NFAT4
E
Relative Bim expression
12
10
8
6
4
2
0
GFP NFAT1 NFAT2 NFAT4

Supplementary Fig. 7

A.

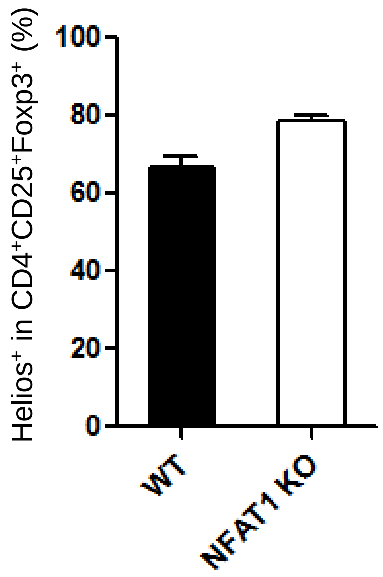


B.

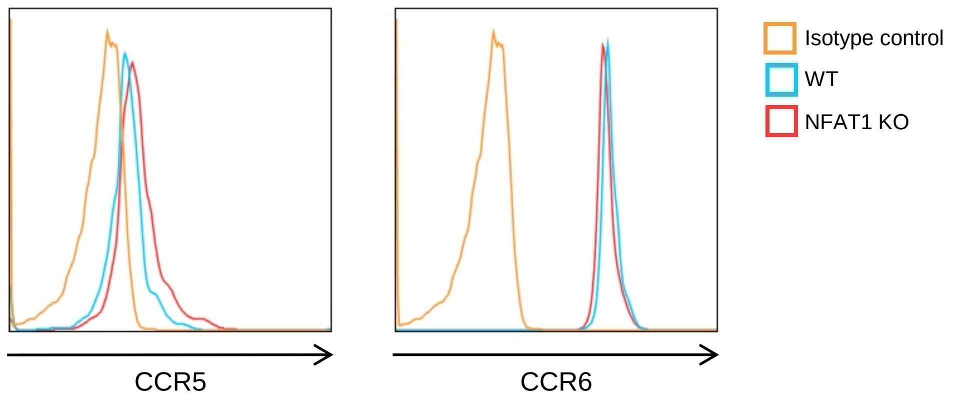


(gated on CD4$^+$CD25$^+$Foxp3$^+$)
